# *EZH2* inactivation in RAS-driven myeloid neoplasms hyperactivates RAS-signaling and increases MEK inhibitor sensitivity

**DOI:** 10.1038/s41375-021-01161-0

**Published:** 2021-02-15

**Authors:** Johannes Lorenz Berg, Bianca Perfler, Stefan Hatzl, Barbara Uhl, Andreas Reinisch, Gudrun Pregartner, Andrea Berghold, Thomas Penz, Michael Schuster, Klaus Geissler, Andreas Prokesch, Carsten Müller-Tidow, Gerald Hoefler, Karl Kashofer, Albert Wölfler, Heinz Sill, Veronica Caraffini, Armin Zebisch

**Affiliations:** 1 Division of Hematology, Medical University of Graz, Graz, Austria; 2 Institute for Medical Informatics, Statistics and Documentation, Medical University of Graz, Graz, Austria; 3 CeMM Research Center for Molecular Medicine of the Austrian Academy of Sciences, Vienna, Austria; 4 5th Medical Department with Hematology, Oncology and Palliative Medicine, Hospital Hietzing, Vienna, Austria; 5 Sigmund Freud University, Vienna, Austria; 6 Gottfried Schatz Research Center for Cell Signaling, Metabolism & Aging, Medical University of Graz, Graz, Austria; 7 Division of Cell Biology, Histology and Embryology, Medical University of Graz, Graz, Austria; 8 BioTechMed-Graz, Graz, Austria; 9 University Hospital Heidelberg, Heidelberg, Germany; 10 Diagnostic and Research Institute of Pathology, Medical University of Graz, Graz, Austria; 11 MRC Cancer Unit, University of Cambridge, Hutchison/MRC Research Centre, Cambridge, United Kingdom; 12 Otto-Loewi Research Center for Vascular Biology, Immunology and Inflammation, Division of Pharmacology, Medical University of Graz, Graz, Austria

**Keywords:** Haematological cancer, Cell signalling

## To the Editor

Mutations modifying *RAS* (*RAS*^*mut*^) occur frequently in myeloid neoplasms (MN) and play a key role in myeloid leukemogenesis [1, 2]. The most commonly observed *RAS*^*mut*^ in MN comprise aberrations in *NRAS* and *KRAS*, as well as in three genes that modulate the levels of active RAS-GTP (*NF1*, *PTPN11*, and *CBL*) [3]. Mechanistically, *RAS*^*mut*^ activate a multitude of downstream signaling cascades, with the MAPK/ERK module being considered one of the major RAS-effector pathways [4]. Consequently, pharmacologic MAPK/ERK inhibition—i.e., by MEK inhibitors—is an appealing therapeutic approach. Indeed, the development of MN in *Ras*^*mut*^ mice can be effectively attenuated by treatment with these substances [2]. Unfortunately, these promising results could not be translated into human MN, with disappointing results in clinical trials [5]. One potential reason is the fact that *RAS*^*mut*^ do not exist as solitary events within these tumors [1, 6]. The existence of co-occurring mutational and non-mutational aberrations has the potential to further influence the activating effects of *RAS*^*mut*^, which ultimately aggravates or inhibits *RAS*^*mut*^-driven leukemogenesis and thereby changes the dependency on activated RAS-signaling [6, 7]. Consequently, these co-occurring events might also change the sensitivity to MEK inhibitors, as recently shown for the co-existence of mutations in *NRAS* and *TET2* [7]. Enhancer of zeste homolog 2 (EZH2) is the core component of the Polycomb Repressive Complex 2 (PRC2). It regulates the expression of a broad range of genes and thereby controls a variety of basic cellular functions [8]. In more detail, EZH2 serves as histone methyltransferase that catalyzes trimethylation of lysine 27 of histone H3 (H3K27me3), which in turn causes the transcriptional repression of its target genes. Inactivation of *EZH2* (*EZH2*^*inact*^)-either by mutation, deletion or a decrease in *EZH2* expression-can be observed in a series of MN [8, 9]. Recently, *EZH2*^*inact*^ has been linked to RAS-signaling as *Ezh2* deletion aggravated the development of *Nras*^*mut*^-driven MN in mice. Moreover, *Ezh2* deletion in *Kras*^*mut*^-induced lung cancers hyperactivated *Kras*^*mut*^-driven MAPK/ERK-signaling [10]. These findings suggest that the dependence on activated RAS-signaling in *RAS*^*mut*^ tumors might be altered by the additional occurrence of *EZH2*^*inact*^.

In this study, we aimed to investigate this hypothesis in the context of myeloid leukemogenesis. By studying almost 450 primary patient specimens with chronic myelomonocytic leukemia (CMML) and acute myeloid leukemia (AML), we show that *EZH2*^*inact*^ and *RAS*^*mut*^ co-exist in MN, and that this co-occurrence is associated with a poor prognosis in affected patients. Importantly, however, we further demonstrate that concomitant *EZH2*^*inact*^ and *RAS*^*mut*^ increases the dependence on RAS-signaling and, consequently, the sensitivity to pharmacologic MEK inhibition in myeloid leukemia cells.

**Fig. 1:**
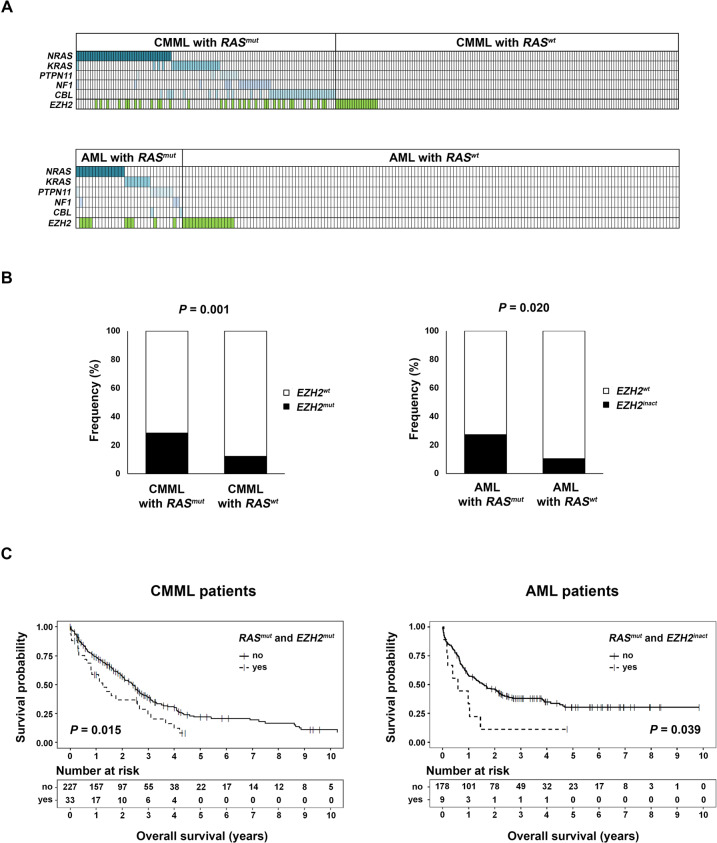
Association between mutations modifying *RAS* and *EZH2* aberrations in MN. **A** Next-Generation Sequencing (NGS) results of 260 chronic myelomonocytic leukemia (CMML) patients studied within the Austrian Biodatabase for CMML [15] showing the distribution of mutations modifying *RAS* (*RAS*^*mut*^; defined as mutations in *KRAS* and *NRAS*, as well as in the RAS-GTP modulators *NF1*, *PTPN11* and *CBL*) and *EZH2*. In summary, 112/260 (43.1%) and 50/260 (19.2%) CMML patients had one or more *RAS*^*mut*^ or *EZH2* mutation(s) (*EZH2*^*mut*^), respectively. Below are the results of the database retrieval of 187 acute myeloid leukemia (AML) patients via The Cancer Genome Atlas (TCGA) [12] showing the distribution of *RAS*^*mut*^ and *EZH2* inactivation (*EZH2*^*inact*^; defined as *EZH2* mutations and/or copy number losses). Every column describes one CMML or AML patient specimen. Colored fields indicate the presence of at least one mutation (for *RAS*^*mut*^) or *EZH2*^*inact*^, respectively. In summary, 33/187 (17.6%) and 25/187 (13.4%) AML patients had one or more *RAS*^*mut*^ mutation(s) or inactivation of *EZH2*, respectively. **B** Within both cohorts, *EZH2* aberrations were significantly more common in patients harboring one or more *RAS*^*mut*^ compared to those without: 28.6%, vs. 12.2% (*P* = 0.001) for the CMML cohort (left), and 27.3%, vs. 10.4% (*P* = 0.020) for the AML cohort (right). Fisher’s exact test was employed for the statistical analysis. **C** Survival curves of the patients belonging to the CMML cohort (left), and the TCGA AML cohort (right). In both cohorts, *RAS*^*mut*^ and *EZH2* aberration co-occurrence was associated with a shortened overall survival (median 14 vs 29 months and 7 vs 19 months for the CMML and AML patients, respectively). Censored events are indicated by a vertical line. A log-rank test was used for these comparisons.

Initially, we were interested whether *EZH2*^*inact*^ and *RAS*^*mut*^ indeed co-exist in MN. Therefore, we re-analyzed previously published Next-Generation Sequencing (NGS) data of 260 chronic myelomonocytic leukemia (CMML) patients within the Austrian Biodatabase for CMML [11]. We chose this entity, since CMML is often driven by mutations modifying the *RAS* genes [1, 3, 6]. Within this cohort, 112/260 (43.1%) patients exhibited at least one *RAS*^*mut*^, *EZH2* was mutated in 50/260 (19.2%) cases and 32/260 (12.3%) presented with both genetic aberrations together. 32/112 (28.6%) *RAS*^*mut*^ patients exhibited additional *EZH2* mutations, whereas 32/50 (64%) cases with *EZH2* mutations presented with an additional *RAS*^*mut*^ (Fig. 1A; Supplementary Table 1). Importantly, the frequency of patients with *EZH2* mutations was increased in cases with one or more *RAS*^*mut*^ (28.6% in *RAS*^*mut*^, vs. 12.2% in *RAS*^*wt*^; *P* = 0.001; Fig. 1B). From a clinical point of view, *RAS*^*mut*^ and *EZH2* aberration co-occurrence was associated with a shortened overall survival (median 14 vs 29 months, *P* = 0.005; Fig. 1C). To delineate whether these findings are of relevance for other MN as well, we then performed a database retrieval of 187 AML patients via The Cancer Genome Atlas (TCGA) (see supplementary methods for details) [12]. In addition to clinical parameters, this database comprises information about mutations, gene expression and DNA copy number variations [12]. Out of the 187 patients within this cohort, 33 (17.6%) exhibited at least one *RAS*^*mut*^, 25/187 (13.4%) exhibited inactivation of *EZH2* and 9/187 (4.8%) presented with both genetic aberrations together. 9/33 (27.3%) *RAS*^*mut*^ patients exhibited with additional *EZH2*^*inact*^, whereas 9/25 (36%) cases with *EZH2*^*inact*^ presented with an additional *RAS*^*mut*^ (Fig. 1A; Supplementary Table 2). Moreover, in line with our data from CMML, *EZH2*^*inact*^ was significantly more common in *RAS*^*mut*^ cases (27.3% in *RAS*^*mut*^ vs. 10.4% in *RAS*^*wt*^; *P* = 0.020; Fig. 1B; *EZH2*^*inact*^ defined as *EZH2* mutations and/or copy number losses). As in CMML, this genetic co-existence was associated with a dismal outcome (median survival 7 vs 19 months, *P* = 0.039, Fig. 1C). Accordingly, the mRNA expression of *EZH2* was significantly decreased in AML cases carrying one or more *RAS*^*mut*^ (Supplementary Fig. 1). Taken together, these data indicate that *RAS*^*mut*^ and *EZH2* aberrations indeed co-exist in human MN and that this co-occurrence seems to be associated with a poor prognosis. Hence, novel therapeutic approaches are desperately needed for these patients, particularly as *RAS*^*mut*^ has been described as difficult to target so far.

**Fig. 2:**
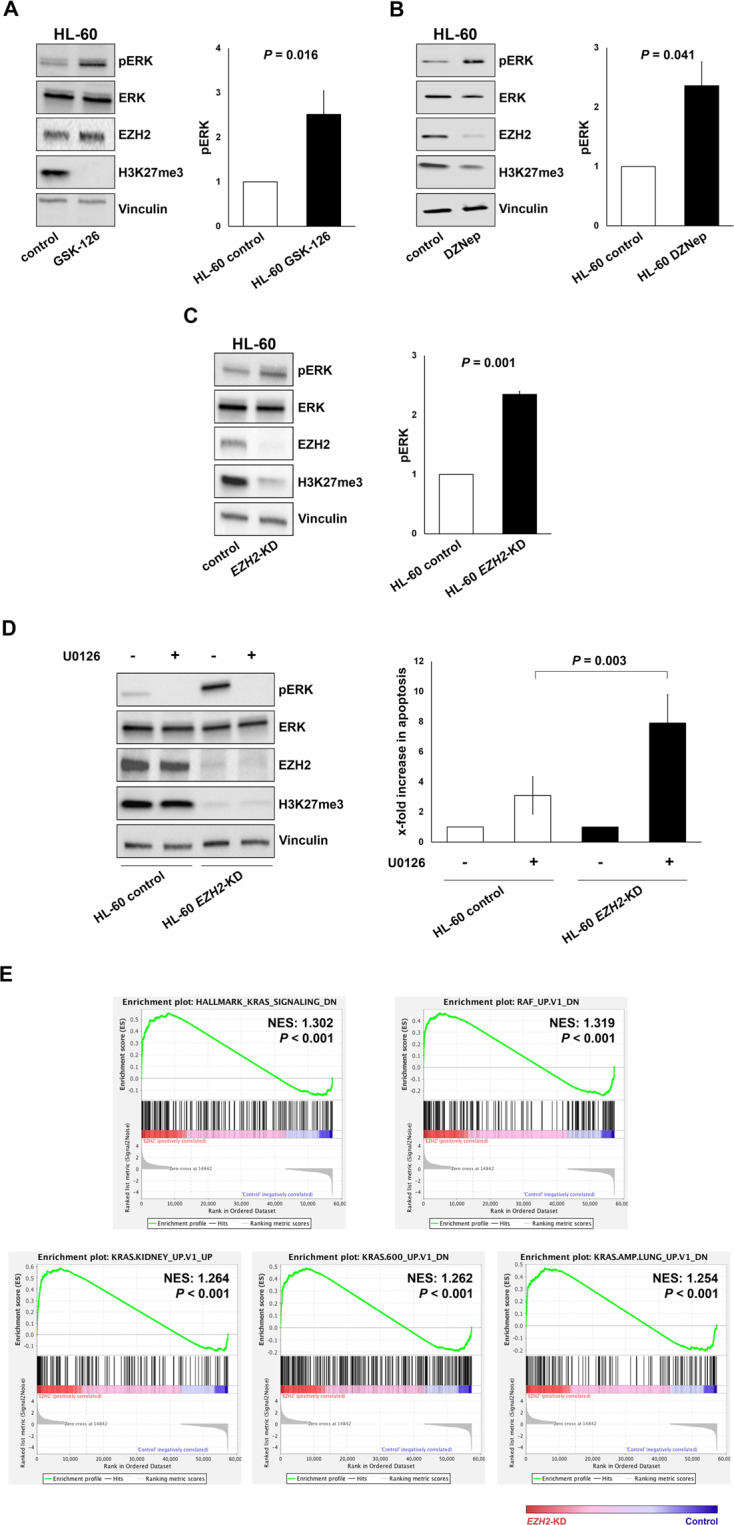
*EZH2* inactivation in *RAS*^*mut*^ myeloid cells amplifies MAPK/ERK-signaling and drives MEK inhibitor sensitivity. The activation of the MAPK/ERK pathway was assessed by the phosphorylation of ERK (pERK) by Immunoblot in HL-60 cells (NRAS Q61L-mutated) after treatment with the EZH2 inhibitors GSK-126 (**A**) and DZNep (**B**). GSK-126 was added at a concentration of 3 µM for 7 days, DZNep at a concentration of 2 µM for 24 h. **C** These experiments were repeated after lentiviral shRNA-mediated *EZH2* knockdown (*EZH2*-KD). The graphs denote the relative increase of pERK expression in the EZH2 inhibitor/KD conditions compared to controls and represent the mean ± standard deviation (SD) of at least three independent experiments. Comparisons against the control condition were performed using a one-sample *t* test against a reference value of 1. **D** HL-60 cells with and without *EZH2*-KD were treated with the MEK inhibitor U0126 (5 µM for 24 h). Subsequently, pERK was assessed by Immunoblot and apoptosis was measured by Annexin-V/7-AAD assay. The graphs denote the x-fold increase in apoptosis in U0126-treated cells compared to the respective vehicle-treated control situation in at least three independent experiments and represent the mean ± SD. Differences between cells with and without *EZH2*-KD were assessed by paired *t* test. **E** Gene set enrichment analysis (GSEA) demonstrating that signatures associated with RAS- and RAF-signaling are enriched within the *EZH2*-KD situation. All signatures displayed exhibited a false discovery rate of below 25%. NES, normalized enrichment score.

Next, we investigated whether *EZH2*^*inact*^ influences the *RAS*^*mut*^-driven MAPK/ERK activation in myeloid leukemia cells (for details on materials and methods see supplementary data). For this purpose, we chose two myeloid cell lines (HL-60 and THP-1). Both carry an activating *RAS*^*mut*^, show normal *EZH2* mRNA expression and lack other *EZH2* aberrations (Supplementary Fig. 2 and Supplementary Table 3). We treated these cells with the two EZH2 inhibitors GSK-126 and 3-Deazaneplanocin A (DZNep), respectively. While GSK-126 is an enzymatic inhibitor, which does not affect EZH2 protein expression itself, DZNep induces EZH2 protein degradation [9]. Both drugs successfully inhibited EZH2 activity, as assessed by reduced H3K27me3 levels. Importantly, however, both inhibitors caused hyperactivation of RAS-MAPK/ERK-signaling, as evidenced by increased phosphorylation of ERK (pERK; Fig. 2A, B; Supplementary Fig. 3A, B). To exclude potential unspecific off-target effects of the EZH2 inhibitors used, we established a puromycin-selected stable short hairpin RNA (shRNA)-mediated *EZH2*-knockdown (*EZH2*-KD) after lentiviral transduction in both cell lines. Empty vector-transduced cells served as controls. Again, *EZH2-*KD reduced H3K27me3 levels and simultaneously increased pERK (Fig. 2C, Supplementary Fig. 4), which indicates that *EZH2*^*inact*^ amplifies MAPK/ERK activation in *RAS*^*mut*^ myeloid cells. Next, we explored whether *EZH2*^*inact*^ increases the sensitivity to MEK inhibitors in *RAS*^*mut*^ myeloid cells. Therefore, we treated HL-60 and THP-1 cells with and without *EZH2-*KD with the MEK inhibitor U0126. U0126 efficiently inhibited pERK in all conditions tested. Most importantly, however, the U0126-induced apoptosis was significantly increased in cells with additional *EZH2-*KD (Fig. 2D; Supplementary Fig. 5), which indicates that these cells are hypersensitive to pharmacologic inhibition of the MAPK/ERK pathway. These findings could be corroborated in 7-AAD/BrdU cell cycle/proliferation assays. Again, the U0126-mediated decrease in proliferation was enhanced in cells with additional *EZH2*-KD (Supplementary Fig. 6). We then aimed to shed more light on the mechanisms behind *EZH2*^*inact*^-induced MAPK-hyperactivation in *RAS*^*mut*^ myeloid cells. As EZH2 regulates a multitude of cellular gene expression profiles via H3K27me3–induced transcriptional repression, we reasoned that *EZH2*^*inact*^ causes the upregulation of genes involved in RAS-MAPK/ERK-signaling. Such a scenario has been identified in a murine in-vivo model of *Kras*^*mut*^*/Ezh2-*deleted lung cancer previously [10]. To test this assumption, we performed RNA-sequencing in HL-60 cells with and without *EZH2-*KD and performed gene set enrichment analysis (GSEA) [13, 14]. Indeed, we observed enrichment of RAS- and RAF-signaling signatures in the *EZH2*-KD situation (Fig. 2E). This included an extensive list of genes that activate the RAS-MAPK/ERK and other signal transduction cascades (Supplementary Table 4).

Finally, we re-analyzed a previously published ChIP-seq dataset of AML cells with *EZH2* loss [9] via the NCBI Gene Expression Omnibus (GSE61785). By focusing on genes with a well-described activator function of RAS-signaling on the one hand, and a significant upregulation in our RNA-seq data of *EZH*-KD cells on the other hand, we were able to demonstrate decreased H3K27me3 signals in the condition with *EZH2* loss (Supplementary Fig. 7). These data suggest that the upregulation of these genes in *EZH2*^*inact*^ cells could indeed be mediated through modification of H3K27me3 within their promoter and/or adjacent genomic regions.

In conclusion, we demonstrate that mutations within genes modifying *RAS* frequently co-occur with inactivation of the epigenetic modifier EZH2 in MN, and that this co-existence is linked to a dismal outcome in affected patients. We further demonstrate that inactivation of *EZH2* amplifies the activation of RAS-MAPK/ERK-signaling in myeloid cells carrying *RAS*-modifying mutations. Most importantly, however, we present preclinical data showing that the co-existence of *EZH2* inactivation and *RAS*-modifying mutations might confer increased sensitivity to MEK inhibitors, thereby providing a potential novel therapeutic rationale for these difficult to treat patients.

## Supplementary information

Supplemental Material
