# Supplementary material for: EZH2 inactivation in RAS-driven myeloid neoplasms hyperactivates RAS-signaling and increases MEK inhibitor sensitivity
Source: Leukemia. 2021 Feb 15;35(5):1521–6. doi: 10.1038/s41375-021-01161-0 (PMC8102185; doi:10.1038/s41375-021-01161-0)
Supplement: Supplementary file 1 — Supplemental Material [file 41375_2021_1161_MOESM1_ESM.pdf]

Supplemental Data

EZH2 inactivation in RAS-driven myeloid neoplasms hyperactivates RAS-signaling and increases MEK inhibitor sensitivity

Berg JL et al.

I) Supplemental Methods

Next-Generation Sequencing

Next-Generation Sequencing (NGS) analysis included a set of genes with recurrent mutations in myeloid neoplasms and was performed on an Illumina MiSeq platform (Illumina Inc, San Diego, CA, USA). Details about the sequencing process and the genes included have been described previously [1]. A variant allele frequency of 5% or higher was considered as positive for analysis.

Cell culture and lentiviral transduction

The cell lines employed in this work were obtained from the German National Resource Center for Biological Material (DSMZ, Braunschweig, Germany). Cells were maintained at 37°C/5% CO_2 in DMEM for 293T HEK packaging cells and RPMI-1640 for HL-60 and THP-1 cells. The medium was enriched with 10% heat-inactivated fetal bovine serum (FBS) and 1X Antibiotic-Antimycotic (Thermo Fischer Scientific, Waltham, MA, USA, including 100U/mL penicillin, 100 mg/mL streptomycin and 0.25 mg/mL amphotericin B). DMEM medium was further supplemented with 1X GlutaMAX (Thermo Fischer Scientific). Of note, cells were serum starved before testing the effects of *EZH2*^{inact} on MAPK/ERK signaling and its biological effects (for details see description of the respective experiments). This previously reported approach[2, 3] was chosen to provide a clean experimental setting, where the effects of *EZH2*^{inact} on *RAS*^{mut}-driven MAPK/ERK activation are not biased by the unphysiologic abundance of growth factors in situations of full serum supplementation. Stocks of cells were regularly re-authenticated in the laboratory by variable number of tandem repeat DNA profiling (VNTR) as previously described [4]. Low passage stocks were frozen and cells were always passaged for less than 6 months after resuscitation.

293T packaging cells were transfected using CalPhos Mammalian Transfection Kit (Clontech, Mountain View, CA, USA) following the manufacturer's instructions. For *EZH2* knockdown (*EZH2*-KD), HL-60 and THP-1 cells were lentivirally transduced with either EZH2 short hairpin RNA (shRNA) or empty control (both psi-LVRU6GP; Genecopoeia, Rockville, MD, USA), respectively. Selection of stable clones for HL-60 and THP-1 cells was performed by adding to the media 0.5 μg/ml and 1 μg/ml puromycin, respectively (Invitrogen, Carlsbad, CA, USA).

Treatment with EZH2 inhibitors

EZH2 inhibition was performed in HL-60 and THP-1 cells using GSK-126 (BioVision, Milpitas, CA, USA) and 3-Deazaneplanocin A (DZNep; Cayman Chemicals, Ann Arbor, MI, USA). GSK-126 was added to the cells at a concentration of 3µM for 7 days in RPMI-1640 media supplemented with 5% FBS. HL-60 and THP-1 cells were treated with 2µM and 1µM of DZNep, respectively, for 24 hours using RPMI-1640 media supplemented with 5% FBS. The same concentration of DMSO was added to the control cells. After the treatment with the inhibitors, cells were collected and used for Immunoblot analysis.

MEK inhibitor treatment; apoptosis and proliferation assays

HL-60 and THP-1 cells were treated with the MEK-inhibitor U0126 (Promega, Madison, WI, USA) at a concentration of 5µM or 10µM for 24 hours, using RPMI-1640 supplemented with 0.05% FBS. Control cells were treated with an equal amount of DMSO. For apoptosis assays, cells were then collected and resuspended in AnnexinV binding buffer (BD biosciences, Franklin Lakes, NJ, USA), stained with AnnexinV and 7-AAD and apoptosis was measured using the CytoFLEX LX flow cytometer (Beckman Coulter, Brea, CA, USA). Proliferation was assessed by Bromodeoxyuridine (BrdU)/ 7AAD flowcytometric assays as previously described [4].

Immunoblot analysis

Ice-cold RIPA-Buffer (Sigma), supplemented with protease and phosphatase inhibitor cocktails (Thermo Fisher Scientific), was added to the HL-60 and THP-1 cell pellets in order to obtain cell lysis. HL-60 *EZH2*-KD and THP-1 *EZH2*-KD cells, as well as the respective control cells, were starved for 24 hours using RPMI-1640 supplemented with 0.05% FBS before lysis. Protein concentration was measured using the DC Protein Assay kit (Bio-Rad, Hercules, CA, USA) following the manufacturer's protocol. Immunoblots were then performed as previously described [3] using Mini-PROTEAN TGX gels for electrophoresis (Bio-Rad) and the Bio-Rad Trans Blot TurboBlotting System for transfer. Polyvinylidene difluoride membranes (Bio-Rad) were incubated with anti-EZH2 (#3147S; Cell Signaling Technologies, Danvers, MA, USA), anti-ERK (#M5670; Sigma-Aldrich, St. Louis, MO, USA), anti-pERK (#4370; Cell Signaling), anti-H3K27me3 (#9733; Cell Signaling) and anti-Vinculin (#ab129002; Abcam, Cambridge, UK). The intensity of the bands was compared using ImageJ [5].

RNA-sequencing

The amount of total RNA was quantified using the Qubit 2.0 Fluorometric Quantitation system (Thermo Fisher Scientific) and the RNA integrity number (RIN) was determined using the Experion Automated Electrophoresis System (Bio-Rad). RNA-sequencing (RNA-seq) libraries were prepared with the TruSeq Stranded mRNA LT sample preparation kit (Illumina) using Sciclone and Zephyr liquid handling workstations (PerkinElmer, Waltham, MA, USA) for pre- and post-PCR steps, respectively. Library concentrations were quantified with the Qubit 2.0 Fluorometric Quantitation system (Life Technologies, Carlsbad, CA, USA) and the size distribution was assessed using the Experion Automated Electrophoresis System (Bio-Rad). For sequencing, samples were diluted and pooled into NGS libraries in equimolar amounts.

Expression profiling libraries were sequenced on HiSeq 3000/4000 instruments (Illumina) in 50-base-pair, single-end mode. Base calls, provided by the real-time analysis (RTA) software (Illumina), were subsequently converted into multiplexed, unaligned BAM format before demultiplexing into sample-specific, unaligned BAM files. For raw data processing off the instruments, custom programs, based on Picard tools (https://broadinstitute.github.io/picard/), were used.

NGS reads were mapped to the Genome Reference Consortium GRCh38 assembly via "Spliced Transcripts Alignment to a Reference" (STAR) [6] utilising the "basic" Ensembl transcript annotation from version e96 (April 2019) as reference transcriptome. Since the hg38 assembly flavour of the UCSC Genome Browser was preferred for alignment and downstream data processing with Bioconductor packages for technical reasons, Ensembl transcript annotation had to be adjusted to UCSC Genome Browser sequence region names before the alignment. STAR was run with options suggested by the ENCODE project. Reads overlapping transcript features were counted with the summarizeOverlaps function of the Bioconductor GenomicAlignments package (https://bioconductor.org/packages/release/bioc/html/GenomicAlignments.html), taking into account that the Illumina TruSeq stranded mRNA protocol leads to sequencing of the second strand so that all reads needed inverting before counting. The Bioconductor DESeq2 [7] package was then used to test for differential expression based on a model using the negative binomial distribution.

An initial exploratory analysis included principal component analysis (PCA), multi-dimensional scaling (MDS), as well as sample distance and expression heatmap plots, all annotated with variables used in the expression modelling (ggplot2 [https://ggplot2.tidyverse.org [8]], Bioconductor ComplexHeatmap - https://bioconductor.org/packages/release/bioc/html/ComplexHeatmap.html - and EnhancedVolcano). Result lists were annotated, filtered for significantly differentially up- and

down-regulated genes and independently subjected to gene set enrichment analysis (Enrichr; https://amp.pharm.mssm.edu/Enrichr/ and https://www.gsea-msigdb.org/gsea/index.jsp) [9, 10]. RNA-seq data have been deposited in the National Center for Biotechnology Information (NCBI)'s Gene Expression Omnibus (GEO; https://www.ncbi.nlm.nih.gov/geo/) and are accessible through GEO accession number GSE150029.

Database retrieval and statistical analyses

EZH2 mRNA expression data were downloaded from The Cancer Genome Atlas AML cohort (TCGA; V2-RNA-seq by Expectation-Maximization [V2 RSEM]) [11], and the Cancer Cell Line Encyclopedia (CCLE; RNA-seq Reads Per Kilobase Million [RPKM]) [12]. Mutations in *NRAS*, *KRAS*, *CBL*, *NF1* and/or *PTPN11*, as well as mutations and/or copy number losses in *EZH2* were downloaded from these cohorts as well. All data were extracted via a database retrieval employing the cBioPortal (https://www.cbioportal.org/) [13, 14]. All data for the TCGA cohort were downloaded 07-APR-2020, data from the CCLE were downloaded 04-FEB-2020.

For ChIP-seq, data from a publicly available dataset[15] were downloaded via the NCBI GEO (https://www.ncbi.nlm.nih.gov/geo/; GSE61785) on 01-OCT-2020. ChIP-seq tracks (wig-files) were imported into the Integrative Genome Viewer suit (https://software.broadinstitute.org/software/igv/). H3K27me3 tracks were displayed together with IgG ChIP tracks and uniformly scaled for each locus.

One-sample t test against a reference value of 1 was employed for the statistical analysis of the Immunoblot results, while paired t test was used for the analysis of the apoptosis assays. Group differences in *EZH2* expression values of primary patients were compared by Mann-Whitney U test, whereas Fisher's exact test was employed for comparison of all dichotomous variables in patient specimens. Kaplan-Meier curves were used to analyze overall patient survival; differences between groups were assessed by log rank test. SPSS (SPSS Inc., Version 25) and

R version 3.6.1 were employed for the analyses and a p value <0.05 was considered statistically significant.

Study approval

The study was approved by the institutional review board of the Medical University of Graz (EK 30-464 ex 17/18) and conducted in agreement with the declaration of Helsinki.

II) Supplemental Figures and Tables

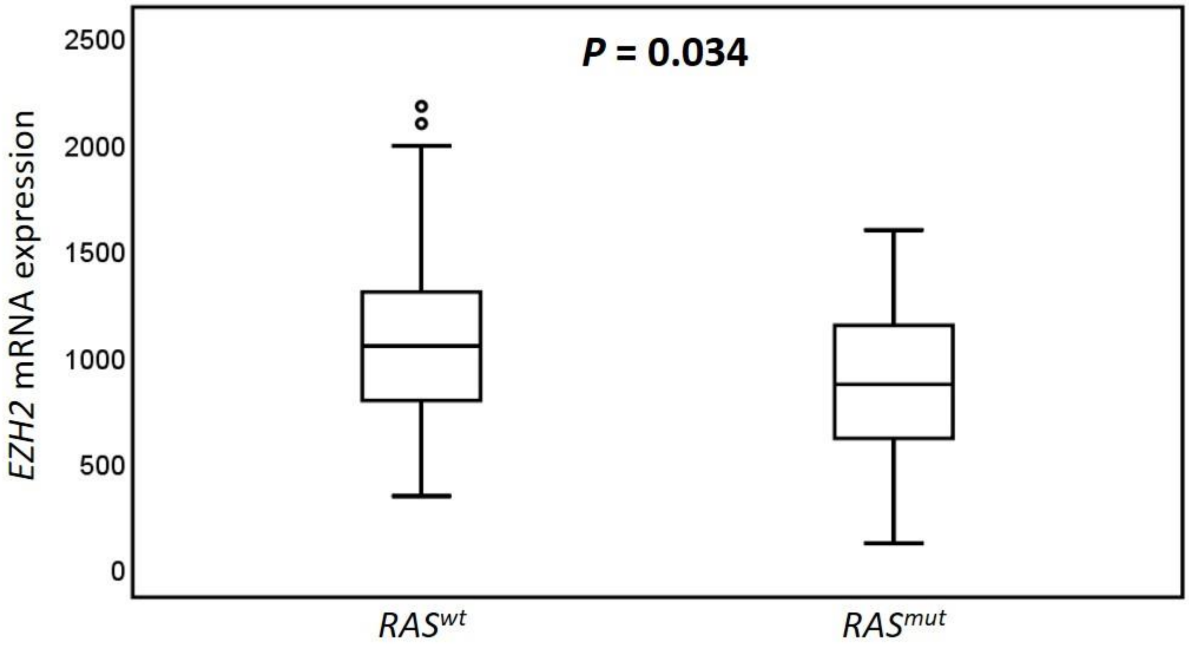

Supplemental Figure 1. mRNA expression of *EZH2* is decreased in *RAS*^{mut} AML patients (TCGA cohort). NGS data for the *RAS*^{mut} status and *EZH2* mRNA expression data were downloaded from The Cancer Genome Atlas AML cohort (TCGA; complete information available in 162 cases) [11]. *EZH2* expression is displayed as V2-RNA-seq by Expectation-Maximization (V2 RSEM). Differences between patients with (n=28) and without (n=134) *RAS*^{mut} were assessed by Mann-Whitney U test.

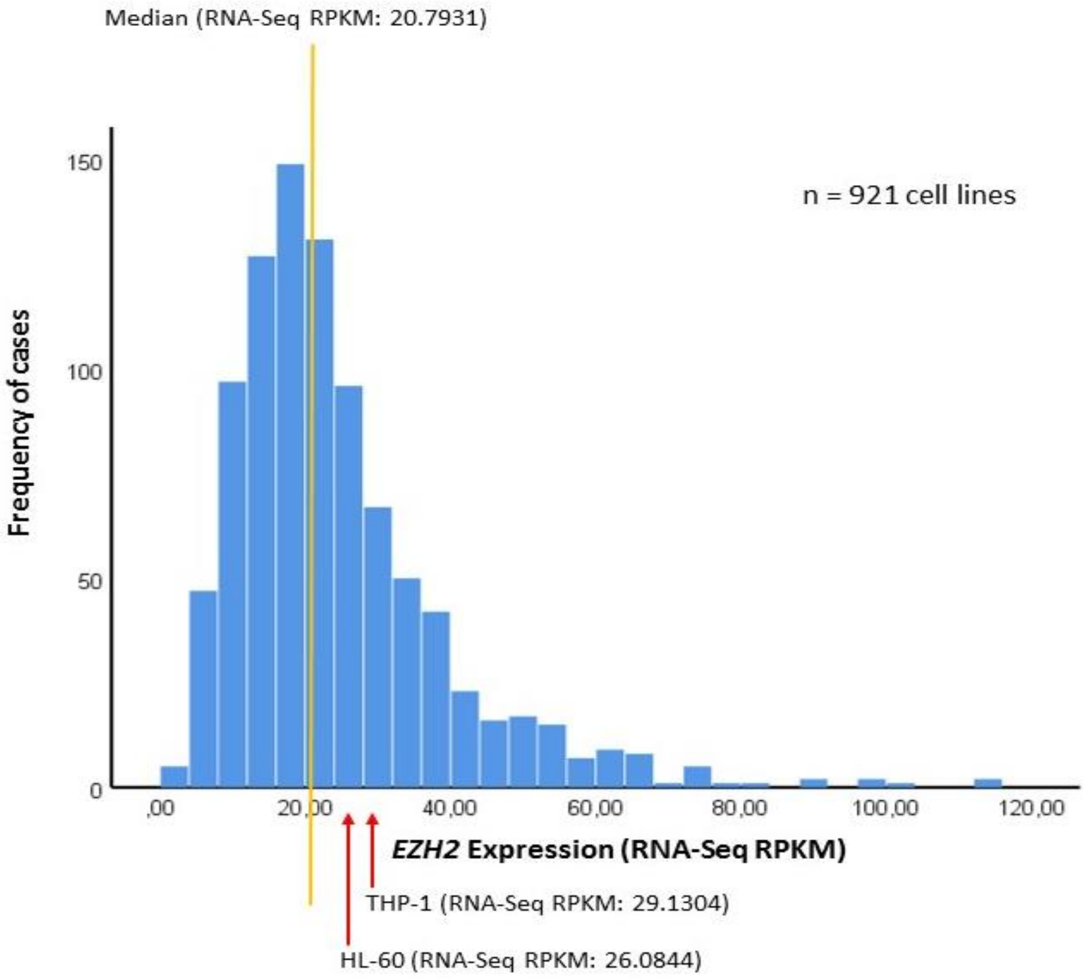

Supplemental Figure 2: *EZH2* mRNA expression is not decreased in HL-60 and THP-1 cells. To exclude *EZH2* inactivation by decreased *EZH2* expression in HL-60 and THP-1 cells, *EZH2* mRNA expression was analyzed within the Cancer Cell Line Encyclopedia [12] via a database retrieval employing the cBioPortal (https://www.cbioportal.org/) [13, 14]. RNA-seq Reads Per Kilobase Million (RPKM) data of 921 cell lines were analyzed and displayed in a histogram. The expression values of HL-60 and THP-1 are close to and above of the median (orange line), which excludes the aberrant expression of *EZH2* within these cell lines.

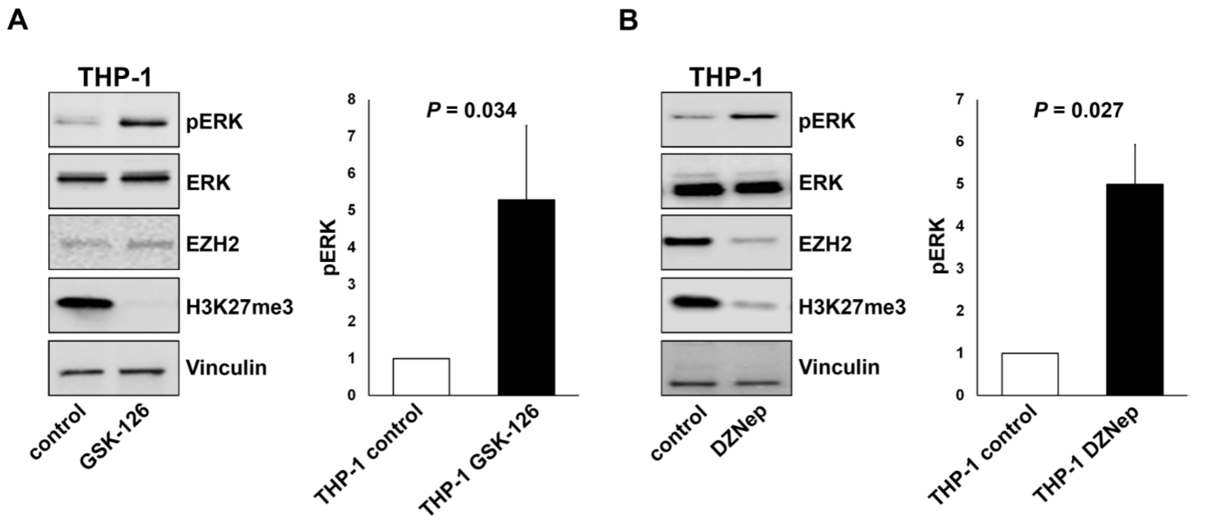

Supplemental Figure 3: Pharmacological *EZH2* inactivation in myeloid cells with mutations modifying *RAS* (*RAS*^{mut}) amplifies MAPK/ERK signaling. The activation of the MAPK/ERK pathway was assessed by the phosphorylation of ERK (pERK) by Immunoblot in THP-1 cells (NRAS G12D-mutated) after treatment with the EZH2 inhibitors GSK-126 **(A)** and DZNep **(B)**. GSK-126 was added at a concentration of 3µM for 7 days, DZNep at a concentration of 1µM for 24 hours. The graphs denote the relative increase of pERK expression in the EZH2-inhibitor conditions compared to controls and represent the mean +/- standard deviation (SD) of at least three independent experiments. Comparisons against the control condition were performed using a one-sample t test against a reference value of 1.

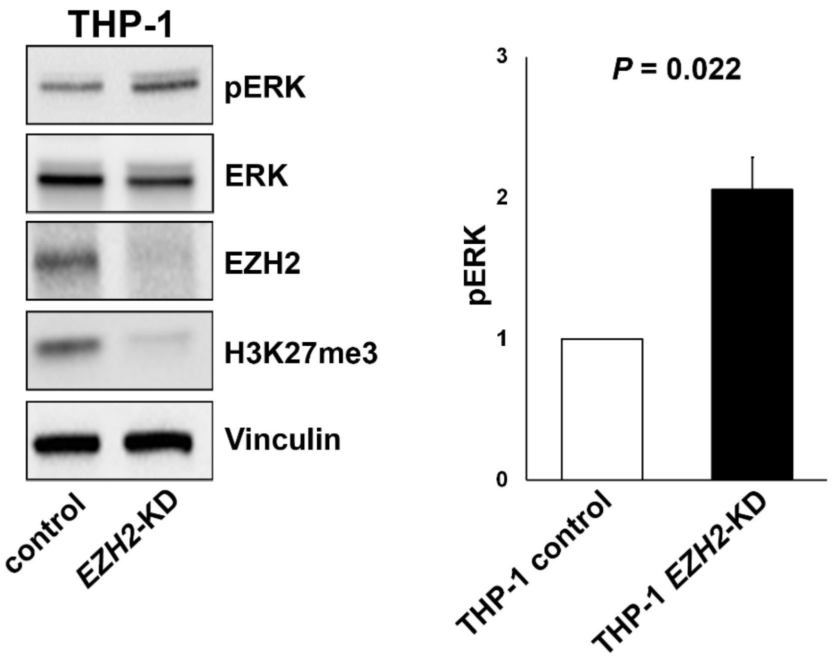

Supplemental Figure 4: *EZH2* inactivation by *EZH2* knockdown in *RAS*^{mut} myeloid cells amplifies MAPK/ERK signaling. The activation of the MAPK/ERK pathway was assessed by the phosphorylation of ERK (pERK) by Immunoblot in THP-1 cells (NRAS G12D-mutated) after lentiviral shRNA-mediated *EZH2* knockdown (*EZH2*-KD). The graph denotes the relative increase of pERK expression in the *EZH2*-KD condition compared to the respective control and represents the mean +/- SD of at least three independent experiments. Comparisons against the control condition were performed using a one-sample t test against a reference value of 1.

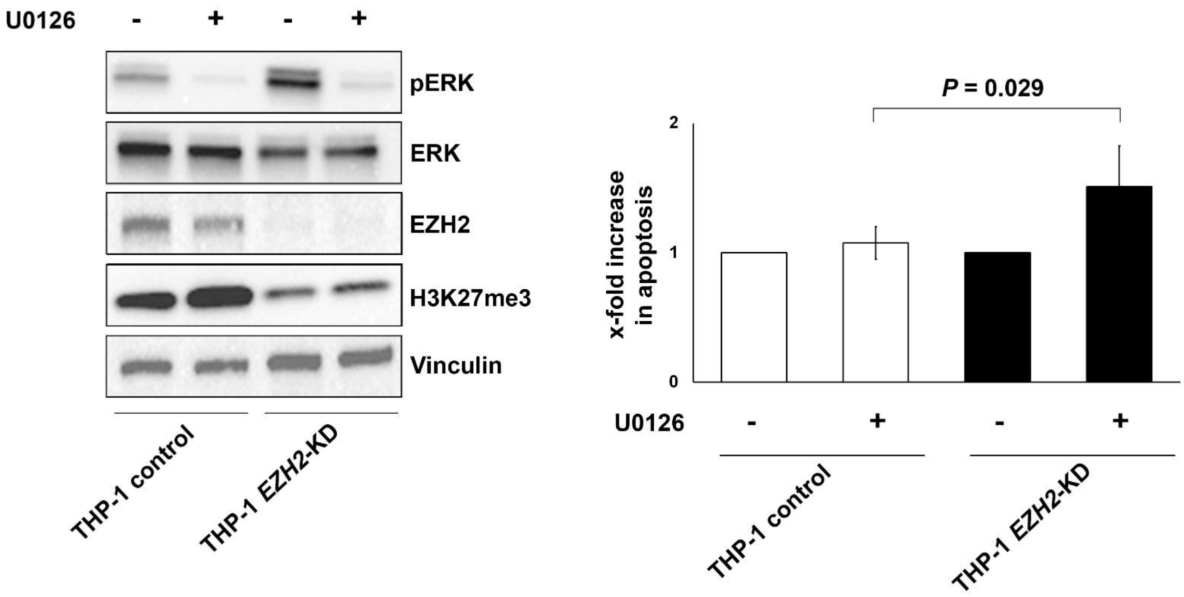

Supplemental Figure 5. ***EZH2*** **inactivation in** ***RAS^{mut}*** **myeloid cells drives MEK-inhibitor sensitivity – effects on apoptosis.** THP-1 cells with and without *EZH2*-KD were treated with the MEK-inhibitor U0126 (5µM for 24 hours). Subsequently, pERK was assessed by Immunoblot and apoptosis was measures by Annexin-V/7AAD assay. The graphs denote the x-fold increase in apoptosis in U0126-treated cells compared to the respective vehicle-treated control situation and represent the mean +/- SD. Differences between cells with and without *EZH2*-KD were assessed by paired t test.

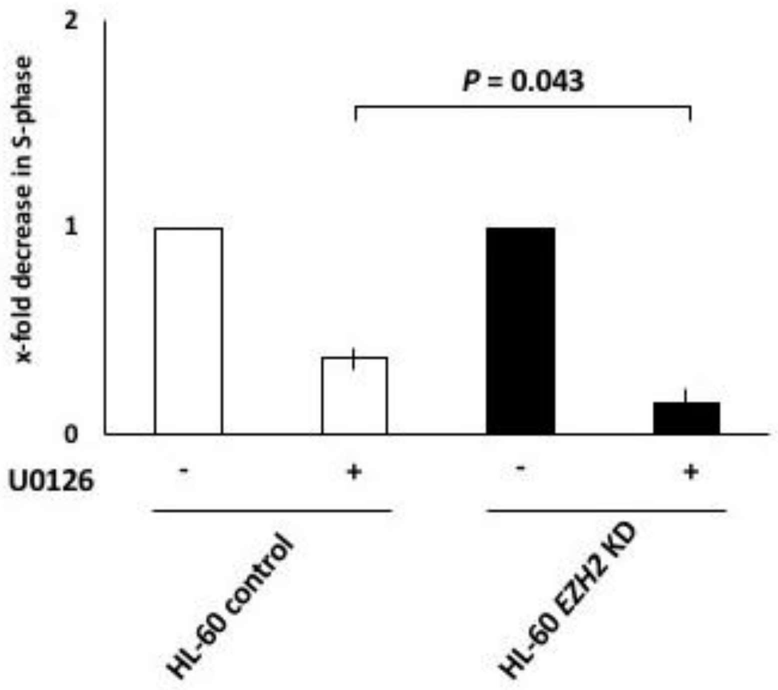

Supplemental Figure 6: *EZH2* inactivation in *RAS*^{mut} myeloid cells drives MEK-inhibitor sensitivity – effects on proliferation. HL-60 cells with and without *EZH2*-KD were treated with the MEK-inhibitor U0126 (10µM for 24 hours). Subsequently, cell cycle/proliferation was measured by BrdU/7-AAD assays. The graphs denote the x-fold decrease in proliferating cells in S-Phase in U0126-treated cells compared to the respective vehicle-treated control situation and represent the mean +/- SD. Differences between cells with and without *EZH2*-KD were assessed by paired t test.

YWHAE (14-3-3ε)

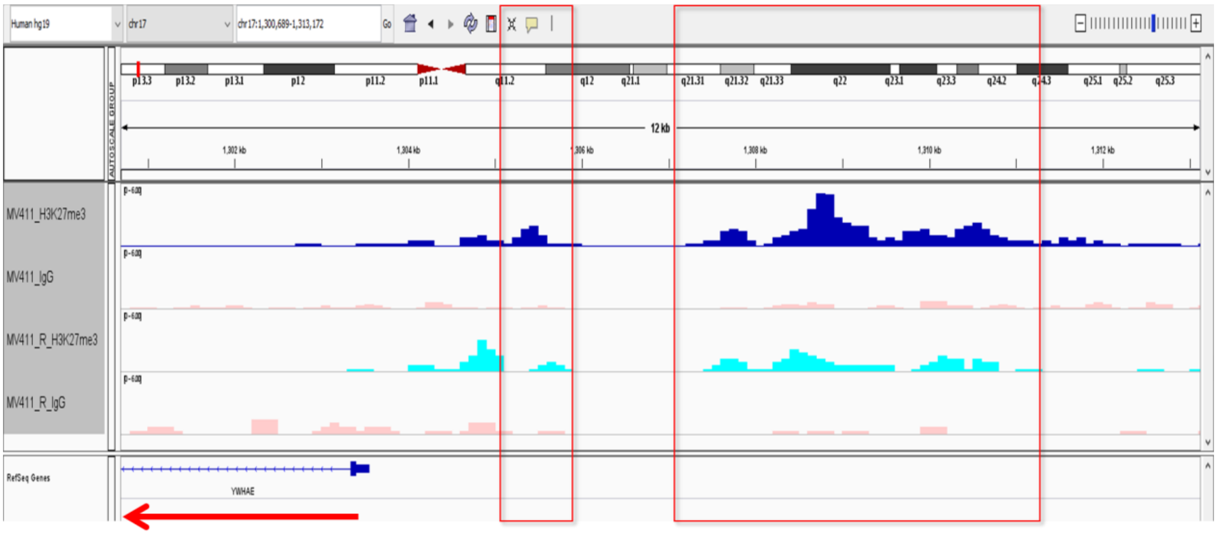

HSP90AA1

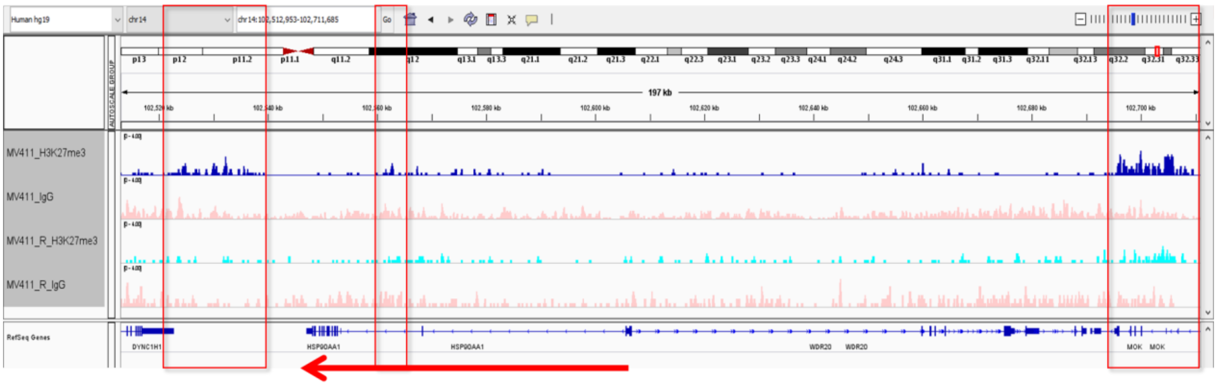

STAT3

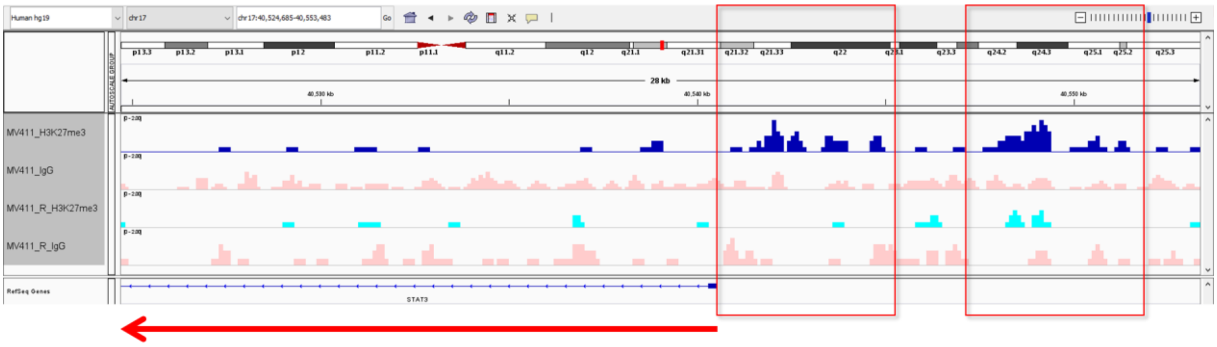

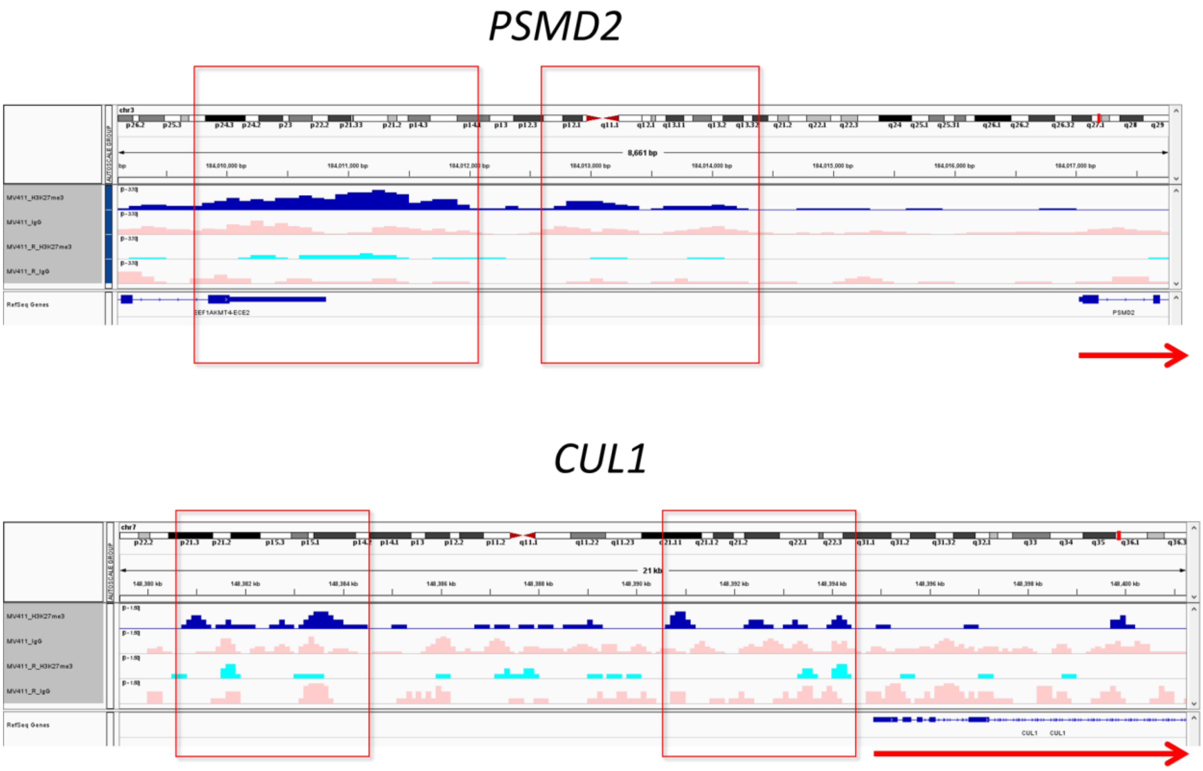

Supplemental Figure 7: The upregulation of RAS-signaling activators in cells with *EZH2* inactivation is associated with decreased H3K27 triple-methylation within their promoter/adjacent genomic regions. H3K27me3 signals in selected promoter and/or adjacent genomic regions was re-analyzed in a publicly available ChIP-seq dataset derived from AML cells with *EZH2* loss (NCBI Gene Expression Omnibus dataset GSE61785).[15] Presented genes were selected based on the fact that they were previously described as activators of RAS-signaling,[16-21] and that they were significantly upregulated in AML cells with *EZH2*-knockdown within our RNA-seq experiments (Supplemental Table 4). These analyses revealed decreased H3K27me3 signals in their promoter and/or adjacent genomic regions in cells with *EZH2* loss (third lane, light blue, MV411_R_H3K27me3) as compared to the wildtype situation (first lane, dark blue, MV411_H3K27me3). The respective isotype controls are displayed in pink. The position and the reading direction of the analyzed genes are indicated by a red arrow. Relevant genomic regions are highlighted by red rectangles. ChIP tracks have been uniformly scaled for each locus.

NRAS	A59D (1); G12A (1); G12D (16); G12R (3); G12V (5); G13D (4); G13V (5); Q61K (2); Y64D (3); Y64N (1)
KRAS	A146T (2); A18D (3); D33E (1); G12C (1); G12D (3); G12R (3); G12S (2); G12V (1); G13C (1); G13D (1); G60V (1); G60_Q61insRL (1); L19F (1); Q22K (1); Q61R (1); T58I (2)
PTPN11	A72T (1); D286Y (1); D61G (1); G503R (1); G93E (1); I96F (1); M504V (2); P144L (1); Q510H (1); V203M (1)
NF1	A2389V (1); D176E (3); E977* (1); F1536S (1); G751V (1); I1641T (1); I2015N (1); I558T (1); L1015P (1); M577I (1); R1477T (1); R2258* (1); R2452C (1); T1184fs (1); V1707D (1); V533F (1); Y2264* (1); Y794C (1); Y1930fs (1)
CBL	C381R (1); C381S (1); C381W (1); C381Y (1); C384Y (1); C404R (1); C404Y (3); C416S (3); C416W (1); C416Y (1); C419S (1); C419Y (1); D390H (1); D390V (1); F418S (2); G413D (1); H398N (1); H398Y (1); K382E (1); L380P (3); P417R (1); P417S (2); R420G (1); R420L (2); R420Q (1); S376P (1); Y371C (1); Y371H (3); E366fs (1); D460del (1)
EZH2	D185H (28); D677G (1); Q553del (1); S371R (1); D730*fs*1 (1); V675M (1); C536F (1); C571Y (1); C695W (1); D659G (1); G660E (1); H501Q (1); K740Sfs (1); M121K (1); N130D (1); N668Y (1); P587fs (1); R288Q (1); R298H (1); R690H (2); S651L (1); T683I (1)

Supplemental Table 1: Mutations identified in the ABCMML dataset. Patients with variations in red were categorized as RAS^{mut} and $EZH2^{inact}$, respectively. In more detail, they fulfilled one of the following criteria: i) the variant has been described in the literature as relevant for the clinical management of myeloid neoplasms (diagnostic, prognostic and/or treatment significance); ii) the variant has been described as pathogenic in functional in-vitro/in-vivo assays; iii) the variant is categorized as "pathogenic" or "likely pathogenic" in the established mutation databases VarSome (https://varsome.com/) and/or COSMIC (Catalogue of Somatic Mutations in Cancer, https://cancer.sanger.ac.uk/cosmic). *NF1* M577I and *CBL* D460del failed to fulfill these criteria. However, the affected patients additionally carried a NRAS G12D (in the case of *CBL* D460del) and CBL H398Y (in the case of *NF1* M577I). Therefore, the patients were still categorized as RAS^{mut}.

Of note, the *EZH2* D185H variation has been described as somatic mutation and single nucleotide polymporphism previously. Importantly, however, functional studies demonstrated impaired EZH2 methyltransferase activity for this substitution, and it was reported as risk factor

for developing malignancies [22-24]. Additionally, D185H was included as relevant substitution in clinical studies of myeloid neoplasms, where *EZH2* mutations were associated with decreased EZH2 protein expression and shortened survival [25]. Therefore, *EZH2* D185H was not excluded from analysis.

NRAS	G12C(1); G12D(3); G13D(5); Q61H(2); Q61K(2); Q61P(1); Q61R(1)
KRAS	G12D(2); G12V(1); G13D(1); I36M(1); A59E(1); Q61H(1); A146T(1)
PTPN11	G60V(1); F71L(1); A72V(1); P491L(1); S502P(1); Q510H(1); Q510L(1); I545L(1)
NF1	NF1(+)LRRC37B(+) fusion (In-frame)(1); R1276Q(1); R1306*(1)
CBL	Q367R(1); X366_splice(1)
EZH2	E740Afs*24 I739Mfs*25(1); R685H(1); X727_splice(1)

Supplemental Table 2: Mutations identified in the TCGA-AML dataset. Patients with variations in red were categorized as *RAS*^{mut} and *EZH2*^{inact}, respectively. In more detail, they fulfilled one of the following criteria: i) the variant has been described in the literature as relevant for the clinical management of myeloid neoplasms (diagnostic, prognostic and/or treatment significance); ii) the variant has been described as pathogenic in functional in-vitro/in-vivo assays; iii) the variant is categorized as "pathogenic" or "likely pathogenic" in the established mutation databases VarSome (https://varsome.com/) and/or COSMIC (Catalogue of Somatic Mutations in Cancer, https://cancer.sanger.ac.uk/cosmic). *NF1*[+]LRRC37B[+] fusion [In-frame] could not be classified as clearly disease-relevant according to these stringent criteria. However, the affected patient carried an additional *NRAS* G12D mutation and was therefore still classified as *RAS*^{mut}.

	Mutations					
	EZH2	*NRAS*	*KRAS*	*PTPN11*	*NF1*	*CBL*
**HL-60**	wt	Q61L	wt	wt	wt	wt
**THP-1**	wt	G12D	wt	wt	wt	wt
	**Copy number**					
	EZH2	*NRAS*	*KRAS*	*PTPN11*	*NF1*	*CBL*
**HL-60**	Diploid	Diploid	Diploid	Diploid	Diploid	Diploid
**THP-1**	Diploid	Diploid	Diploid	Diploid	Diploid	Diploid

Supplemental Table 3: Absence of mutations and/or copy number aberrations of *EZH2* in HL-60 and THP-1. Mutations and copy number aberrations were analyzed in the *RAS*-modifying genes *NRAS*, *KRAS*, *CBL*, *NF1*, and *PTPN11*, as well as in *EZH2* within the Cancer Cell Line Encyclopedia [12]. These analyses demonstrate the absence of *EZH2* mutations and copy number alterations in both cell lines. Both cell lines harbor an activating hotspot mutation in *NRAS*.

Category	Upregulated Genes
**PI3K-Akt Signaling Pathway (WP4172)**	*CDKN1A;TGFA;PIK3CB;THBS3;RPTOR;FGF5;PPP2CB;CCND2;PPP2R3C;MYC;AKT2;MYB;AKT1;THEM4;HSP90AA1;MAP2K2;ANGPT1;HGF;RPS6;OSM;RBL2;G6PC3;CCNE1;RHEB;CDC37;PPP2R2D;BCL2;SGK1;TLR4*
**Ras Signaling (WP4223)**	*RALA;MAP2K2;RAB5C;RASA4B;RALB;PLA2G2C;PIK3CB;RASGRP2;PLD1;ETS2;RASA3;RASA4;AKT2;AKT1;ABL2;RAC3;CALM3;CALM1;PRKACB;RALGDS;PAK4*
**EGF/EGFR Signaling Pathway (WP437)**	*VAV3;STAT5B;RALA;MAP2K2;RALB;SH3KBP1;STAT3;PEBP1;FOS;PLD1;PIK3C2B;DOK2;RPS6KA5;HGS;RPS6KA2;GRB10;AKT1;FOSB;SPRY2;ARHGEF1;RALGDS;CRK*
**MAPK Signaling Pathway (WP382)**	*ARRB1;ECSIT;RASGRP2;FGF5;RPS6KA5;PPP3CC;DUSP10;MYC;AKT2;CASP3;MKNK2;AKT1;RAC3;FLNB;PRKACB;MAP3K7;MAP2K3;MAP2K4;DAXX;DUSP2;MAP2K2;GADD45A;CACNA2D1;CACNA2D4;FOS;MAPK8IP2;MAPK12;CDC25B;MAPK13;NR4A1;PPP5C;CACNB3;FAS;TAB1;MAP3K14;CRK*
**ErbB Signaling Pathway (WP673)**	*MAP2K4;STAT5B;CDKN1A;MAP2K2;TGFA;PIK3CB;MYC;AKT2;AKT1;ABL2;CRK;HBEGF;PAK4*
**PDGF Pathway (WP2526)**	*NFKBIA;MAP2K4;STAT3;ARFIP2;WASL;FOS*
**p38 MAPK Signaling Pathway (WP400)**	*MAP2K4;DAXX;RPS6KA5;TRADD;MYC;MAP3K7*
**VEGFA-VEGFR2 Signaling Pathway (WP3888)**	*YWHAE;CXCL8;MAPKAP1;TXN;SHB;NDRG1;ICAM1;CAMKK2;HDAC7;RPS6KA5;ADAMTS1;RACK1;GRB10;AKT1;FLNB;WASF1;MAP2K3;EGR1;MAP2K4;HSP90AA1;JAG1;MAP2K2;STAT3;RPS6;LIMK1;PLAUR;NFATC2;F3;MAPK12;NFKBIA;NR4A1;HGS;AKT1S1;BCL2;CTNNB1;FAS;PRKD2;CRK;HBEGF*
**TGF-beta Signaling Pathway (WP366)**	*CDKN1A;CUL1;TERT;MYC;AKT1;E2F5;MAP3K7;JUNB;MAP2K3;TGIF1;MAP2K4;TRAP1;MAP2K2;SMURF2;WWP1;FOS;PJA1;RBL2;DAB2;ZEB2;ZFYVE16;TFDP1;HGS;STRAP;FOSB;SIK1;TAB1*
**IL-2 Signaling Pathway (WP49)**	*STAT5B;CCND2;MAP2K2;CISH;MYC;STAT3;RPS6;BCL2;AKT1;FOS*
**AMP-activated Protein Kinase (AMPK) Signaling (WP1403)**	*CDKN1A;CPT1A;PFKFB3;PRKAG1;PRKAG2;PIK3CB;EEF2;ADIPOR2;CAMKK1;CAMKK2;RPTOR;AKT2;FASN;LEPR;AKT1;PRKACB*
**Wnt Signaling Pathway (WP363)**	*GSK3A;CTBP1;LEF1;LRP5;NFATC2;CSNK1D;CSNK1E;MYC;DVL1;AKT1;CTNNB1;PIP5K1B;TCF3;MAP3K7*
**Hedgehog Signaling Pathway (WP4249)**	*SMURF2;PTCH1;PTCH2;ARRB1;CSNK1D;CSNK1E;GRK3;CCND2;SUFU;GPR161;BCL2;PRKACB;CSNK1G2*
**TNF alpha Signaling Pathway (WP231)**	*TRADD;CUL1;PYGL;TXN;CASP3;PSMD2;AKT1;RFFL;BID;MAP3K7;GLUL;MAP2K3;MAP2K4;TRAP1;HSP90AA1;CSNK2A1;CYBA;TRAF1;NFKBIA;CDC37;BAX;TAB1;MAP3K14;NSMAF;NFKBIB*

Supplemental Table 4: Genes upregulated in HL-60 cells with *EZH2* knockdown. The genes associated with RAS-MAPK/ERK signalling, which are upregulated in HL-60 cells harboring *EZH2* knockdown, are listed in this table. The genes were obtained using Gene set enrichment analysis (WikiPathways_2019_Human; Enrichr; https://amp.pharm.mssm.edu/Enrichr/).

Supplemental References:

1. Geissler K, Jager E, Barna A, Gurbisz M, Marschon R, Graf T, *et al*. The Austrian biodatabase for chronic myelomonocytic leukemia (ABCMML) : A representative and useful real-life data source for further biomedical research. *Wien Klin Wochenschr* 2019; **131**: 410-8.

2. Caraffini V, Geiger O, Rosenberger A, Hatzl S, Perfler B, Berg JL, *et al*. Loss of RAF kinase inhibitor protein is involved in myelomonocytic differentiation and aggravates RAS-driven myeloid leukemogenesis. *Haematologica* 2020; **105**: 375-86.

3. Zebisch A, Wolfler A, Fried I, Wolf O, Lind K, Bodner C, *et al*. Frequent loss of RAF kinase inhibitor protein expression in acute myeloid leukemia. *Leukemia* 2012; **26**: 1842-9.

4. Hatzl S, Geiger O, Kuepper MK, Caraffini V, Seime T, Furlan T, *et al*. Increased Expression of miR-23a Mediates a Loss of Expression in the RAF Kinase Inhibitor Protein RKIP. *Cancer Res* 2016; **76**: 3644-54.

5. Schneider CA, Rasband WS, Eliceiri KW. NIH Image to ImageJ: 25 years of image analysis. *Nat Methods* 2012; **9**: 671-5.

6. Dobin A, Davis CA, Schlesinger F, Drenkow J, Zaleski C, Jha S, *et al*. STAR: ultrafast universal RNA-seq aligner. *Bioinformatics* 2013; **29**: 15-21.

7. Love MI, Huber W, Anders S. Moderated estimation of fold change and dispersion for RNA-seq data with DESeq2. *Genome Biol* 2014; **15**: 550,014-0550-8.

8. Wickham H. ggplot2: Elegant Graphics for Data Analysis. 2016;: 260.

9. Mootha VK, Lindgren CM, Eriksson KF, Subramanian A, Sihag S, Lehar J, *et al*. PGC-1alpha-responsive genes involved in oxidative phosphorylation are coordinately downregulated in human diabetes. *Nat Genet* 2003; **34**: 267-73.

10. Subramanian A, Tamayo P, Mootha VK, Mukherjee S, Ebert BL, Gillette MA, *et al*. Gene set enrichment analysis: a knowledge-based approach for interpreting genome-wide expression profiles. *Proc Natl Acad Sci U S A* 2005; **102**: 15545-50.

11. Cancer Genome Atlas Research Network. Genomic and epigenomic landscapes of adult de novo acute myeloid leukemia. *N Engl J Med* 2013; **368**: 2059-74.

12. Ghandi M, Huang FW, Jane-Valbuena J, Kryukov GV, Lo CC, McDonald ER,3rd, *et al*. Next-generation characterization of the Cancer Cell Line Encyclopedia. *Nature* 2019; **569**: 503-8.

13. Gao J, Aksoy BA, Dogrusoz U, Dresdner G, Gross B, Sumer SO, *et al*. Integrative analysis of complex cancer genomics and clinical profiles using the cBioPortal. *Sci Signal* 2013; **6**: pl1.

14. Cerami E, Gao J, Dogrusoz U, Gross BE, Sumer SO, Aksoy BA, *et al*. The cBio cancer genomics portal: an open platform for exploring multidimensional cancer genomics data. *Cancer Discov* 2012; **2**: 401-4.

15. Gollner S, Oellerich T, Agrawal-Singh S, Schenk T, Klein HU, Rohde C, *et al*. Loss of the histone methyltransferase EZH2 induces resistance to multiple drugs in acute myeloid leukemia. *Nat Med* 2017; **23**: 69-78.

16. Banerji U. Heat shock protein 90 as a drug target: some like it hot. *Clin Cancer Res* 2009; **15**: 9-14.

17. Brachet-Botineau M, Polomski M, Neubauer HA, Juen L, Hédou D, Viaud-Massuard MC, *et al*. Pharmacological Inhibition of Oncogenic STAT3 and STAT5 Signaling in Hematopoietic Cancers. *Cancers (Basel)* 2020; **12**: 240. doi: 10.3390/cancers12010240.

18. Fischer A, Baljuls A, Reinders J, Nekhoroshkova E, Sibilski C, Metz R, *et al*. Regulation of RAF activity by 14-3-3 proteins: RAF kinases associate functionally with both homo- and heterodimeric forms of 14-3-3 proteins. *J Biol Chem* 2009; **284**: 3183-94.

19. Kalkman HO. Potential opposite roles of the extracellular signal-regulated kinase (ERK) pathway in autism spectrum and bipolar disorders. *Neurosci Biobehav Rev* 2012; **36**: 2206-13.

20. Ren ZQ, Yan WJ, Zhang XZ, Zhang PB, Zhang C, Chen SK. CUL1 Knockdown Attenuates the Adhesion, Invasion, and Migration of Triple-Negative Breast Cancer Cells via Inhibition of Epithelial-Mesenchymal Transition. *Pathol Oncol Res* 2020; **26**: 1153-63.

21. Zhang Z, Li H, Zhao Y, Guo Q, Yu Y, Zhu S, *et al*. Asporin promotes cell proliferation via interacting with PSMD2 in gastric cancer. *Front Biosci (Landmark Ed)* 2019; **24**: 1178-89.

22. Burgos S, Montalban-Bravo G, Fuente L, Jabbour EJ, Kanagal-Shamanna R, Soltysiak KA, *et al*. Novel EZH2 mutation in a patient with secondary B-cell acute lymphocytic leukemia after deletion 5q myelodysplastic syndrome treated with lenalidomide: A case report. *Medicine (Baltimore)* 2019; **98**: e14011.

23. Cohen AS, Yap DB, Lewis ME, Chijiwa C, Ramos-Arroyo MA, Tkachenko N, *et al*. Weaver Syndrome-Associated EZH2 Protein Variants Show Impaired Histone Methyltransferase Function In Vitro. *Hum Mutat* 2016; **37**: 301-7.

24. Li H, Chang C, Shang Y, Qiang L, Zhang B, Bu B, *et al*. A missense variant in EZH2 is associated with colorectal cancer risk in a Chinese population. *Oncotarget* 2017; **8**: 94738-42.

25. McGraw KL, Nguyen J, Al Ali NH, Komrokji RS, Sallman D, Zhang X, *et al*. Association of EZH2 protein expression by immunohistochemistry in myelodysplasia related neoplasms with mutation status, cytogenetics and clinical outcomes. *Br J Haematol* 2019; **184**: 450-5.
